# A Case of Metastatic Pancreatic Adenocarcinoma in Complete Remission Using Chemotherapy and Immunotherapy

**DOI:** 10.7759/cureus.13133

**Published:** 2021-02-04

**Authors:** Saad Atiq, Osman O Atiq, Zainab O Atiq, Syed Samad, Omar Atiq

**Affiliations:** 1 Internal Medicine, Duke University Medical Center, Durham, USA; 2 Internal Medicine, Unity Health System, Searcy, USA; 3 Internal Medicine, University of Arkansas for Medical Sciences, Little Rock, USA; 4 Internal Medicine, St. Matthew’s University School of Medicine, George Town, CYM; 5 Hematology and Oncology, University of Arkansas for Medical Sciences, Little Rock, USA

**Keywords:** immunotherapy, pembrolizumab, complete remission, pancreatic adenocarcinoma

## Abstract

Among the various types of cancer, pancreatic cancer is considered to have a particularly grim prognosis. Treatment includes surgery, chemotherapy, or both. While the role of immunotherapy is well-studied in many types of cancer, such is not the case with pancreatic cancer.

A 49-year-old female presented to the oncology clinic following a biopsy of a pancreatic mass. CT-guided needle biopsy of the mass demonstrated moderately differentiated pancreatic adenocarcinoma. Positive emission tomography-computed tomography (PET-CT) revealed metastases to the liver. She was started on chemotherapy with FOLFIRINOX (leucovorin calcium, 5-fluorouracil, irinotecan hydrochloride, oxaliplatin) and demonstrated over 60% reduction in the size of liver metastases within three months. PET-CT four months after initiation of chemotherapy revealed no focal avid fluorodeoxyglucose (FDG) uptake in the liver, and the pancreatic body mass was stable in size at 3.0 cm with stable standardized uptake value (SUV) max at 2.4, only slightly elevated from 1.9 on the previous scan. Further treatment with chemotherapy was halted after 18 cycles due to side effects. With the patient’s tumor being epidermal growth factor receptor (EGFR) negative, mismatch repair (MMR) negative, 3% tumor cells PD-L1 positive with 10% tumor-associated immune cells positive, treatment with pembrolizumab was started. Follow-up PET-CTs over the next several months confirmed the patient was in complete remission from metastatic pancreatic cancer. At the time of the report, the patient had a durable response of three years.

We report a rare case of complete remission of metastatic pancreatic adenocarcinoma treated with chemotherapy, followed by immunotherapy. With emerging targets for modification of tumor microenvironment, immunotherapy must be further explored in the treatment of pancreatic cancer.

## Introduction

Cancer remains one of the deadliest diseases both within the United States and globally. Among the various types of cancer, pancreatic cancer is considered to have a particularly grim prognosis, with five-year survival rates ranging from 2% to 9% [1]. Risk factors include but are not limited to age, obesity, diabetes, smoking, alcohol, chronic pancreatitis, family history, and prior abdominal radiotherapy [2]. Diagnosis can be made using CT, positive emission tomography-computerized tomography (PET-CT), endoscopic ultrasound, magnetic resonance cholangiopancreatography (MRCP), endoscopic retrograde cholangiopancreatography (ERCP), and percutaneous biopsy [3]. Significant advances have been made in the treatment of many cancers, particularly with the development of immunotherapy and more targeted radiation. Pancreatic cancer, however, remains difficult to treat, due to its aggressive nature, lack of well-studied screening tools, and limited treatment options. Although ineffective as a screening tool, CA 19-9 is a marker often used to monitor response to treatment [1]. Mainstays for treatment depend on the type of pancreatic cancer, as well as its location and regional involvement, but generally involve surgery, chemotherapy, radiation, or a combination of the three. While the role of immunotherapy is well-studied in many types of cancer, such is not the case with pancreatic cancer. Herein, we present a case of a patient with metastatic pancreatic adenocarcinoma, now in complete remission after treatment with chemotherapy, then pembrolizumab.

## Case presentation

**Figure 1:**
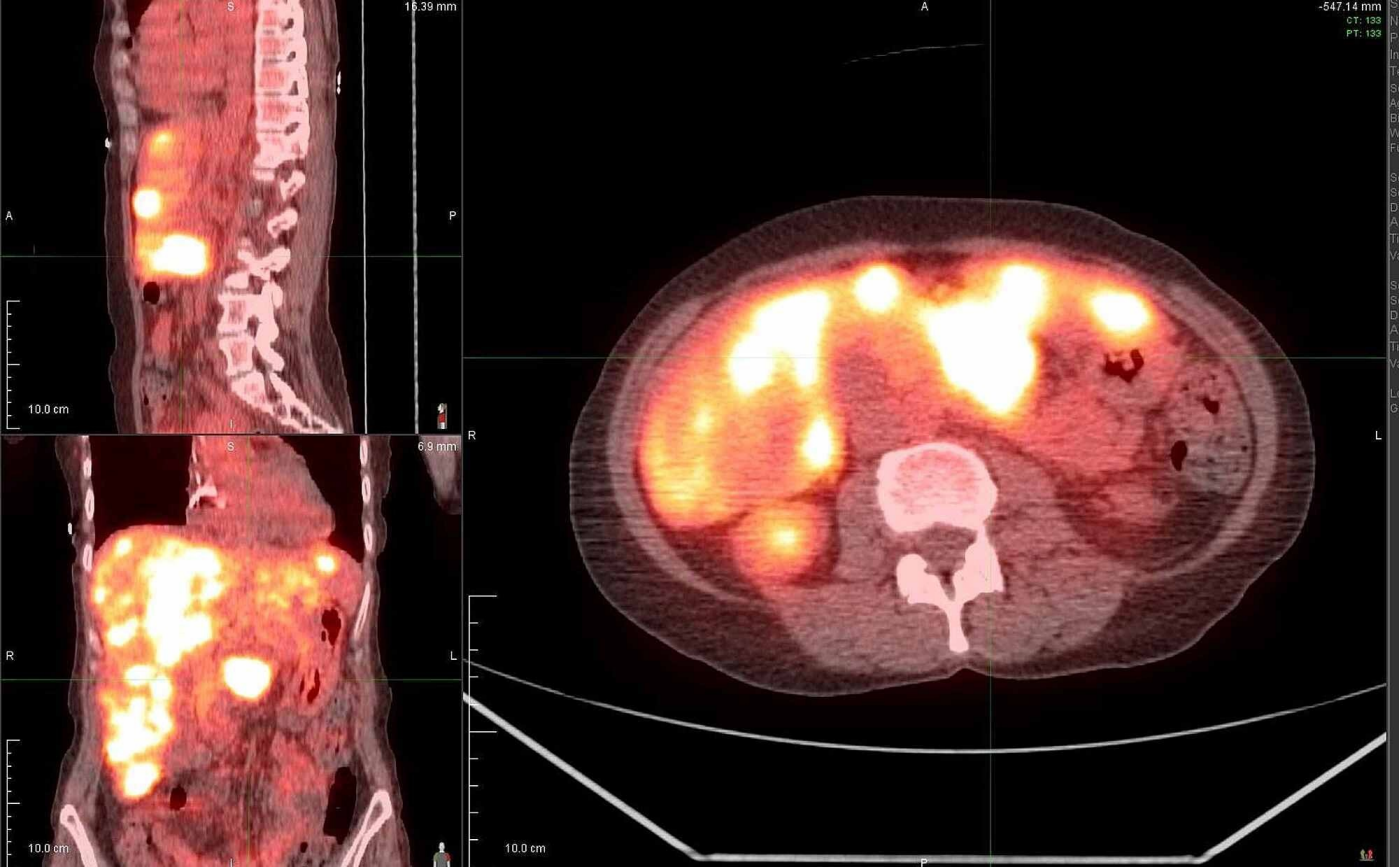
PET-CT demonstrating pancreatic adenocarcinoma with metastases to the liver, prior to the initiation of treatment

**Figure 2:**
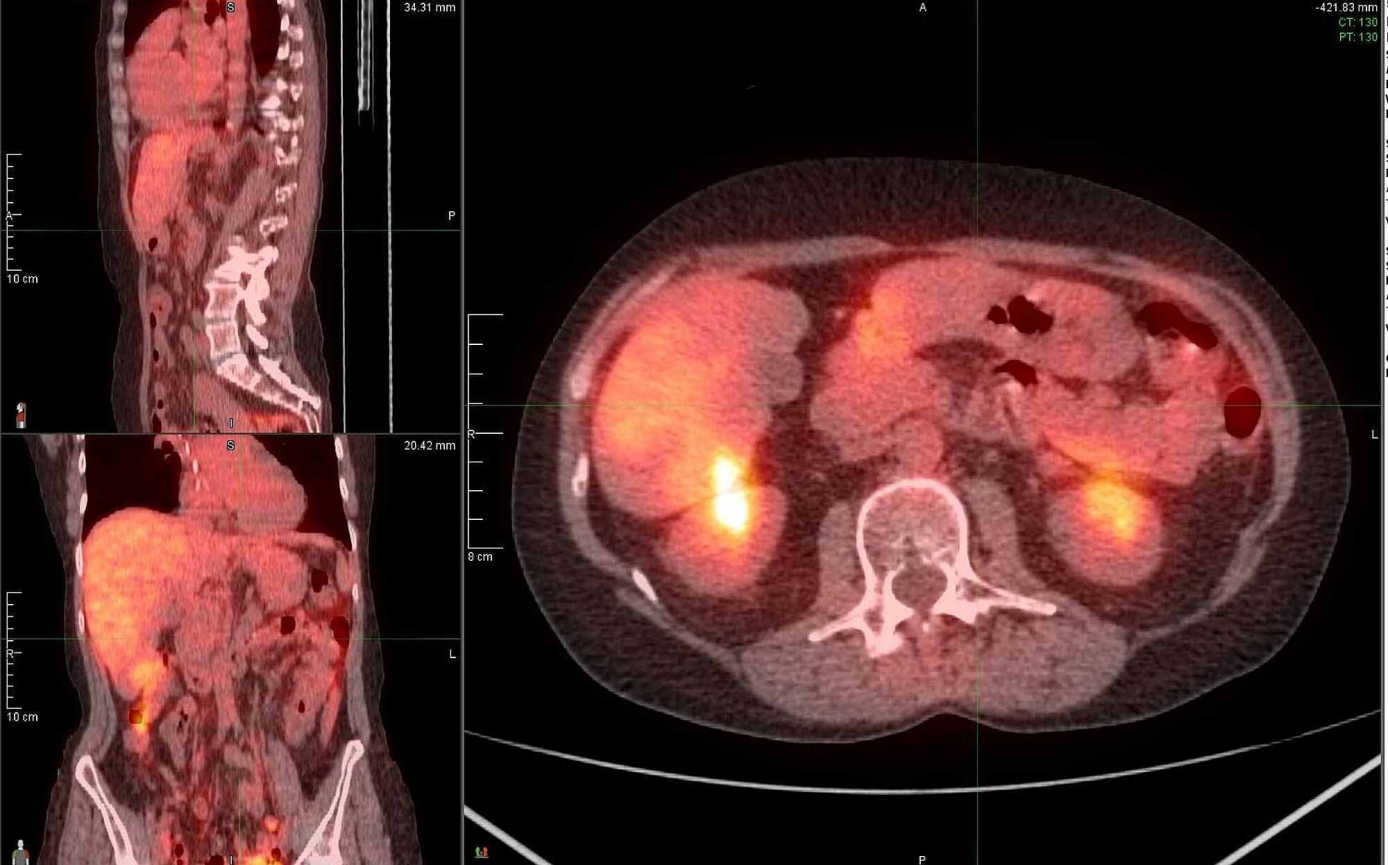
PET-CT demonstrating complete remission two months after starting treatment with pembrolizumab

A 49-year-old female with a history of alcohol abuse, tobacco abuse, and hypertension, presented to the oncology clinic as a referral following biopsy of a pancreatic mass. She began having right upper quadrant abdominal pain, mid-back pain, changes in bowel habits, and abdominal bloating three months prior, and she noticed a 5-pound weight loss during this time frame. CT scan of the abdomen revealed a 3.3 cm mass in the body of the pancreas, and subsequent CT-guided needle biopsy of the mass demonstrated moderately differentiated pancreatic adenocarcinoma. PET-CT revealed metastases to the liver (Figure 1). She was started on chemotherapy with FOLFIRINOX (leucovorin calcium, 5-fluorouracil, irinotecan hydrochloride, oxaliplatin) and demonstrated over 60% reduction in the size of liver metastases within three months. PET-CT at six months showed hepatic fluorodeoxyglucose (FDG) uptake only mildly increased from the background and stable size of pancreatic mass with FDG uptake below background activity. Baseline CA 19-9 was obtained at nine months and measured 13 (reference range <35 U/mL). One month later, CA 19-9 level was 10. PET-CT revealed no focal avid FDG uptake in the liver, and the pancreatic body mass was stable in size at 3.0 cm with a stable standardized uptake value (SUV) max at 2.4, only slightly elevated from 1.9 on the previous scan. After 18 cycles of chemotherapy, additional treatment options were explored as the patient had received near maximum benefit from adjuvant therapy and was experiencing worsening side effects including progressive peripheral neuropathy. The patient’s tumor was epidermal growth factor receptor (EGFR) negative, mismatch repair (MMR) negative, 3% tumor cells PD-L1 positive with 10% tumor-associated immune cells positive. Given the patient's young age and excellent functional status, approval for treatment with pembrolizumab was obtained and therapy was started. Prior to treatment, CA 19-9 level was nine. Two months later, this level had decreased to six, and CT abdomen one month later after four cycles of pembrolizumab showed no evidence of disease. Follow-up PET-CTs over the next several months confirmed the patient was in complete remission from metastatic pancreatic cancer (Figure 2). At the time of the report, the patient had a durable response of three years.

## Discussion

Among the various types of pancreatic cancer, pancreatic adenocarcinoma is the most common, accounting for nearly 85% of all diagnoses [1]. In early-stage cases without local invasion, surgical resection with adjuvant chemotherapy is the treatment of choice. With the local invasion, neo-adjuvant chemotherapy with either FOLFIRINOX or nab-paclitaxel plus gemcitabine has become standard, followed by a re-evaluation for surgical resection. However, more than 50% of cases are identified after metastases, and chemotherapy in these patients confers poor survival rates [4]. Median survival in patients with advanced pancreatic cancer is less than 12 months despite the treatment options available [5].

With such poor outcomes, the need for new therapies is evident. Immunotherapy is being investigated given its success with other forms of cancer, and pembrolizumab was recently approved for the treatment of advanced pancreatic cancers [6]. It has been noted that some forms of cancer will respond to checkpoint inhibitors regardless of whether or not the tumor expresses PD-L1. Furthermore, PD-L1 expression can be variable even within the tissue of a single tumor [7]. PD-L1 expression in pancreatic cancer has been associated with worse clinical outcomes as well, again highlighting this interaction as a potential therapy target [8]. The phase II COMBAT trial showed a potential role for PD-1 blockade in combination with chemokine receptor 4 (CXCR4) blockade [9]. However, many early-phase clinical trials with checkpoint inhibitors have not shown success in treating pancreatic adenocarcinoma, which is suspected to be due to the tumor microenvironment and its relative lack of effector T cells [8]. Modifying the tumor microenvironment to allow for the cytotoxic effect of T cells may be a useful approach towards improving the efficacy of immunotherapy. Pre-clinical studies in mice have shown an improved response of tumors treated concurrently with PD-1/PD-L1 blockade and gemcitabine [10]. Studies of mice models showed that fibroblast activation protein (FAP)-positive stromal cells that were initially depleted were able to respond to treatment with immunotherapy [11]. Furthermore, mice with pancreatic ductal adenocarcinoma and overexpression of the tumor suppressor ETS homologous factor (EHF) were more responsive to anti-PD1 therapy, underscoring the importance of the tumor microenvironment in treatment response [12]. While Ca 19-9 is often used to monitor treatment response, new studies have revealed a role for using leukemia inhibitory factor (LIF) as a protein marker to observe disease progression and response to treatment [13]. Additionally, deletion of the gene for LIF in mouse models showed enhanced chemosensitivity and slowed disease progression [13]. Thus, LIF may be a potential target to modify the pancreatic tumor microenvironment that can improve the response to immunotherapy as well.

Expanding upon the success of pancreatic adenocarcinoma treatment with immunotherapy in mouse trials, this case demonstrates the need for further investigation of immunotherapy as a treatment modality for pancreatic cancer. It is possible the prior treatment with FOLFIRINOX created a favorable tumor microenvironment prior to the initiation of immunotherapy. With emerging targets for modification of tumor microenvironment, including FAP-positive stromal cells and LIF, immunotherapy must be further explored as a treatment option for pancreatic cancer.

## Conclusions

This appears to be the only reported case in the literature of complete remission of metastatic pancreatic adenocarcinoma treated with chemotherapy, followed by immunotherapy. Furthermore, the durable response of three years is remarkable in what is widely considered a fatal prognosis. Prior treatment with FOLFIRINOX may have created a favorable tumor microenvironment preceding immunotherapy. With emerging targets for modification of tumor microenvironment, including FAP-positive stromal cells, EHF, and LIF, the role of immunotherapy in the treatment of pancreatic cancer must be further explored.
